# Supplementary material for: Identification of potential biomarkers in dengue via integrated bioinformatic analysis
Source: PLoS Negl Trop Dis. 2021 Aug 4;15(8):e0009633. doi: 10.1371/journal.pntd.0009633 (PMC8336846; doi:10.1371/journal.pntd.0009633)
Supplement: Attachment — Submitted filename: Response to Reviewers.docx [file pntd.0009633.s001.docx]

Dear Editor

Sincerest thanks for your response and reviewers’ comments on our manuscript entitled " *Identification of potential biomarkers in dengue via integrated bioinformatic analysis*" (PNTD-D-20-02220). We have revised the manuscript and would like to resubmit it for your consideration. We have carefully studied the comments made by the reviewers and amended the manuscript. And the revised manuscript has been submitted. Point by point responses to the reviewers’ comments are listed below this letter.

We hope that the revised version of the manuscript is now acceptable for publication in your journal.

I am looking forward to hearing from you soon.

With best wishes,

Yours sincerely,

Xu-Guang Guo.

We would like to express our sincere thanks to the reviewers for the constructive and positive comments.

**Replies to Reviewer 1:**

**Methods**

**-Are the objectives of the study clearly articulated with a clear testable hypothesis stated?**

**-Is the study design appropriate to address the stated objectives?**

**-Is the population clearly described and appropriate for the hypothesis being tested?**

**-Is the sample size sufficient to ensure adequate power to address the hypothesis being tested?**

**-Were correct statistical analysis used to support conclusions?**

**-Are there concerns about ethical or regulatory requirements being met?**

1. **ALL CRITERIA ARE MET FOR METHODS.**

**Answer:** We are very grateful to the reviewers for reviewing our article and making positive comments. Your affirmation is extremely valuable to us, and we are greatly encouraged.

**Results**

**-Does the analysis presented match the analysis plan?**

**-Are the results clearly and completely presented?**

**-Are the figures (Tables, Images) of sufficient quality for clarity?**

2. **All criteria are met.**

**Answer:** Thank you very much for your comment and recognition.

**Conclusions**

**-Are the conclusions supported by the data presented?**

**-Are the limitations of analysis clearly described?**

**-Do the authors discuss how these data can be helpful to advance our understanding of the topic under study?**

**-Is public health relevance addressed?**

3. **The conclusions are ok except for the microRNA part , which is not done correctly to predict microRNAs as biomarkers for deng.**

**Answer:** Our team has discussed the microRNA part, and confirmed the opinions of the reviewers. Because we also considered that after all the microRNAs are predicted based on the interaction with key genes and miRNA with miRTarBase database, not the direct analysis of the original data. Therefore, the microRNA part was not considered as potential markers in this study.

**Editorial and Data Presentation Modifications?**

**Use this section for editorial suggestions as well as relatively minor modifications of existing data that would enhance clarity. If the only modifications needed are minor and/or editorial, you may wish to recommend “Minor Revision” or “Accept”.**

4. **It is ok.**

**Answer:** Thank you for your reading and comment carefully. Your recognition is a great encouragement to us.

**Summary and General Comments**

**Use this section to provide overall comments, discuss strengths/weaknesses of the study, novelty, significance, general execution and scholarship. You may also include additional comments for the author, including concerns about dual publication, research ethics, or publication ethics. If requesting major revision, please articulate the new experiments that are needed.**

5. **The study is ok; but with no new findings for pathogenesis of deng discovered except cell cycle which is already known, and the microRNA sectis not done rightlyion.**

**Answer:** Thank the reviewers for their comments and affirmation on our research. We modified the study according to the questions raised by the reviewers. We also modified the content of microRNA section. We updated the prediction results and did not use it as one of the markers of this study.

**Replies to Reviewer 2:**

1. **Are the objectives of the study clearly articulated with a clear testable hypothesis stated?**

**- The objective of the study is to identify dengue infection biomarkers. In order to do so the authors analyze public data with a standard methodology.**

**Answer:** Thank you very much for your comment and recognition.

2. **Is the study design appropriate to address the stated objectives?**

**- Yes, it is. By analyzing available transcriptomes, filtering DEGs, proceeding with pathway enrichment and then looking for miRNAs, the authors are able to find potential biomarkers for dengue infection. However, without at least an in vitro experiment in KO or of some sort targeted pathways in cell line DENV-infected x control in order to validate what was found, the findings in this publication are susceptible to criticism.**

**Answer:** We are truly grateful for your reading and comment carefully. However, due to the lack of financial support and laboratory limitations, it is difficult for us to carry out any in vitro experiments or some targeted pathway of DENV infection in X control group to verify what we have found. However, we also hope that through the relevant bioinformatics studies, we can provide some directional guidance for the future research of dengue infection, and contribute our strength to the public health.

3. **Is the population clearly described and appropriate for the hypothesis being tested?**

**- In fact, no. All three of the datasets chosen for this study have data from different timepoints of infection (post symptoms onset). During infections and diseases progression, patients’ transcriptional profile is in constant change and dengue infection is no exception. In fact, dengue infection is known to exhaust T and B lymphocytes (present in PBMCs, the samples from these studies) during initial stages of the infection but this changes in the convalescent stage. Since the transcriptional profile of each stage is so different, which of the samples were used and compared? There are no mentions to this point in the manuscript.**

**Answer:** First of all, we would like to express our heartfelt thanks to the reviewers for their detailed comments! In fact, we also recognize that the transcriptional profiles of patients are constantly changing during infection and disease progression, and dengue fever infection is no exception. We carefully reviewed the samples we adopted and found that the problem was indeed what the reviewers said. Therefore, we grouped dengue patients according to the different time points after the symptoms of infection, and only adopted the samples of patients with fever and the samples of the control group. We have to admit that after grouping, we have got very different results. Therefore, we revised most of the results and discussion part, and we also added the corresponding description on the material and method of the specific sample situation.

4. **Is the sample size sufficient to ensure adequate power to address the hypothesis being tested?**

**- Yes, the datasets combined have enough samples to ensure adequate power if all the samples are used. However, I do not believe a transcriptional profile from a convalescent patient would be equal from a patient in the initial stages of the infection. Since the study is trying to find biomarkers, by analyzing all of this data together the statistical tests should be removing potential DEGs from the initial stages just because they are not differentially expressed two weeks later. That being the case, should the authors take this into consideration, I cannot guarantee there are enough samples in these three datasets.**

**Answer:** Thank you very much for your comments. According to the reviewers' opinions, we reviewed the samples used in this study and only included the samples of fever stage. The GSE28405 dataset was composed of 26 control samples and 31 dengue infection samples in fever stage, while the GSE38246 and GSE51808 were composed of 8, 9 control samples and 105, 28 fever infection samples, respectively. So we think that to some extent, the samples of three datasets are still enough for analysis.

5. **Were correct statistical analysis used to support conclusions?**

**- Yes, both limma and GEO2R are bioinformatics’ tools that are already published and are highly accepted by scientists.**

**Answer:** Thank you very much for your comment and recognition.

6. **Are there concerns about ethical or regulatory requirements being met?**

**- No, there are not.**

**Answer:** Thank you for reading and comment carefully.

7. **Does the analysis presented match the analysis plan?**

**- Yes, it does.**

**Answer:** Thank you very much for your comment and recognition.

8. **Are the results clearly and completely presented?**

**- Yes, they are.**

**Answer:** Thank you very much for your comment and recognition.

9. **Are the figures (Tables, Images) of sufficient quality for clarity?**

**- Yes, they are.**

**Answer:** Thank you very much for your comment and recognition.

10. **Are the conclusions supported by the data presented?**

**- Yes**

**Answer:** Thank you very much for your comment and recognition.

11. **Are the limitations of analysis clearly described?**

**- No**

**Answer:** We have added a description of limitations in the discussion section according to the situation of this study, which can be seen in the penultimate paragraph of the discussion section.

12. **Do the authors discuss how these data can be helpful to advance our understanding of the topic under study?**

**- Yes, both in the introduction and in the conclusion**

**Answer:** Thank you very much for your careful comment and recognition.

13. **Is public health relevance addressed?**

**- Yes, identifying potential biomarkers for disease treatment (and in this case, infection control) are of extreme importance. Even more when talking about dengue, its relevance goes without saying given the number of infected people yearly.**

**Answer:** We are truly grateful for your reading and comment carefully.

14. **Editorial and Data Presentation Modifications?**

**Use this section for editorial suggestions as well as relatively minor modifications of existing data that would enhance clarity. If the only modifications needed are minor and/or editorial, you may wish to recommend “Minor Revision” or “Accept”.**

**I strongly suggest an English revisor in case the paper is accepted due to some English mistakes. Text without numerated lines so it is hard to reference, but some examples in the introduction are:**

**"There is a few dengue vaccine but no specific antiviral treatment" -> are … vaccines**

**"A DENV vaccine can not elicits protection" -> cannot elicit**

**"mainstream view is that immunity leads to cytokine storm cytokine storm" -> repetition**

**"many patients with DENV infection without developing plasma leakage" -> do not develop**

**"which is meaningful to predict sever dengue" -> severe**

**Answer:** We are very sorry for these writing mistakes and have corrected in the article. In addition, with the help of English professionals, we used the relevant grammar checking online tools to review the full text.

15. **Also, there is an inconsistency (probably a typo in the abstract?):**

**"Through overlapping, a total of 66 differentially expressed genes (DEGs) were identified, of which 53 were upregulated and 24 were downregulated." (results 3.1 show 13 downregulated, 53+24 do not add up to 66)**

**Answer:** We are really sorry for the math mistake. This problem has been revised and the latest results were showed in the abstract section.

16. **Summary and General Comments**

**As stated before, a differentiation in samples from different time (days) since symptoms onset is needed. Since transcriptional profiles are distinct, in order to find better biomarkers it would be required to at least not group convalescent blood samples with initial infection samples. Also, an in vitro experiment in cell lineage (jukarT, B-LCL, ...) in which the identified miRNA were targeted (either via KO by CRISPR/Cas) or maybe the mRNAs were silenced by siRNA would also be interesting. But i also understand that since no funding is linked to this research, the in vitro experiment may be out of question.**

**Answer:** First of all, we would like to thank the reviewers for their careful review and positive comments. We also noticed that the development of the disease is different at different times (days). Therefore, after reviewing the whole study and discussion, we divided the patients into groups according to the different periods of the samples collected, and only the samples of patients with fever were adopted by our study. In addition, we are also very grateful for the understanding of the reviewers. Without financial support, it is really difficult for us to carry out in vitro experiments. However, we still hope that our research results can provide some help for the research field of dengue infection partly.

**Replies to Reviewer 3:**

1. **Methods**

**-Are the objectives of the study clearly articulated with a clear testable hypothesis stated?**

**-Is the study design appropriate to address the stated objectives?**

**-Is the population clearly described and appropriate for the hypothesis being tested?**

**-Is the sample size sufficient to ensure adequate power to address the hypothesis being tested?**

**-Were correct statistical analysis used to support conclusions?**

**-Are there concerns about ethical or regulatory requirements being met?**

**Yes the objectives are clearly stated, still few things are lacking in the manuscript.**

**Yes the sample size is good enough to address the hypothesis.**

**1) As all the three gene expression data come from same disease condition but other factors are highly varying. The authors should explain in detail the significance and preselection condition for these datasets to consider in the study.**

**Answer:** We have to admit that although these three gene expression data are from the same disease condition, other factors are quite different. Before obtaining the data sets, we searched the GEO database for dengue fever, and then according to the relevant conditions, in order to eliminate the difference factors to the greatest extent, we grouped the GSE28405, GSE38246 and GSE51808 datasets according to the different time points of dengue fever patients. Considering the consistency of the samples, we only used the samples and control samples taken in the fever period In this paper, we will make further bioinformatics analysis.

**2) The author should state which method were used for data normalization.**

**Answer:** We used the R 4.0.1 statistical software (https://www.r-project.org/) and a Bioconductor (http://bioconductor.org/biocLite.R) to process raw data and screen differentially expressed genes. The data of GSE28405 and GSE38246 were batch calibrated and standardized by using the *limma* package. The data of GSE51808 was batch calibrated and standardized by using the *affy* package. The related description has been added in *Data processing* section of the *Materials and methods.*

**3) What is the rational for selection of fold change value?**

**Answer:** According to statistical knowledge, as we all know, P < 0.05 shows that the difference is statistically significant. At the same time, fold change is the difference multiple of sample quality inspection expression, and its value needs to be > 1. Generally, fold change will be taken as 2. However, considering that it can ensure significant differences and screen out more differential genes for further analysis and comparability before data sets, this study took p < 0.05 and FC ≥ 1.5 when analyzing each dataset. Because when fold change took 2, the number of DEGs obtained from individual data sets are relatively small.

2. **Results**

**-Does the analysis presented match the analysis plan?**

**-Are the results clearly and completely presented?**

**-Are the figures (Tables, Images) of sufficient quality for clarity?**

**1) Yes, The analysis is presented matches the analysis plan**

**Answer:** We are very grateful to the reviewers for reviewing our article and making positive comments. Your affirmation as extremely valuable to us, and we are greatly encouraged.

**2) In results section**

**Figure 5 is missing**

**Figure 5c is missing**

**Figure 6 an 7 description is missing in the text.**

**Answer:** First of all, we would like to express our sincere regret for the mistakes in these details on the chart. Since we re-analyzed the data and got the new results, we have replaced our results and re-described our new results in this paper. For these errors, we carefully repeated the comparison to ensure that the same error does not happen again. Once again, I felt sorry for this and thanking for reviewers' careful examination.

**3) In discussion part references are missing.**

**The author either missed or overlooked some parts in the results and discussion, it should be carefully checked and edited.**

**Answer:** We’re verry sorry for our carelessness. We found missing and added the corresponding description in the results and discussion. In terms of the reference missing in discussion, we also added some articles after reviewing the article and reference list. Through combining your valuable suggestions, we researched and reviewed the references in discussion. We are very sorry for the mistake and have corrected in the article.

3. **Conclusions**

**-Are the conclusions supported by the data presented?**

**-Are the limitations of analysis clearly described?**

**-Do the authors discuss how these data can be helpful to advance our understanding of the topic under study?**

**-Is public health relevance addressed?**

1) **Yes the conclusion is well supported by the data presented, limitation of the study is clearly stated.**

**Answer:** We are very grateful to the reviewers for reviewing our article and making positive comments. Your affirmation as extremely valuable to us, and we are greatly encouraged.

2) **The author should add few points relating to future use of outcome in the conclusion section.**

**Answer:** Thank you very much for your comments, we have added that our study may have potential implications for future prediction of disease progression in symptomatic dengue patients, and has important significance for the pathogenesis and targeted therapy of dengue in the last paragraph.

**3) The author should corelate the study with public health importance in the conclusion section**

**Answer:** Thank you very much for your comments, we have added the status of dengue fever and the importance of public health in conclusion.

4. **Editorial and Data Presentation Modifications?**

**Use this section for editorial suggestions as well as relatively minor modifications of existing data that would enhance clarity. If the only modifications needed are minor and/or editorial, you may wish to recommend “Minor Revision” or “Accept”.**

**Major revision is needed.**

**(As above stated points are significant to the study design)**

**Answer:** Thank you for your comments. We have made major revisions. At the same time, I hoped you can comment and consider our article carefully again.

5. **Summary and General Comments**

**Use this section to provide overall comments, discuss strengths/weaknesses of the study, novelty, significance, general execution and scholarship. You may also include additional comments for the author, including concerns about dual publication, research ethics, or publication ethics. If requesting major revision, please articulate the new experiments that are needed.**

**The study is a good and scientifically sound. Biomarkers are an important biological components in diagnosis and identification of disease and its severity. The DENV infection in tropical and sub-tropical region are big challenges to the health department. Identification of potential biomarkers will help us in diagnosis and identification of drug targets.**

**The current approach is insilico based analysis hence a clinical validation is needed.**

**Answer:** Thank you very much for your reading and comment carefully. We also believe that future research is needed to make up for our current limitations, and we hope that future research can have updated progress. We hope that the final version submitted by us can meet the requirements of the journal! If you still have any questions, please contact us!

We tried our best to improve the manuscript and made some changes in the manuscript. We appreciate for Editor/Reviewers’ warm work earnestly, and hope that the correction will meet with approval. Once again, thank you very much for your comments and suggestions.
