# Supplementary material for: Identification of potential biomarkers in dengue via integrated bioinformatic analysis
Source: PLoS Negl Trop Dis. 2021 Aug 4;15(8):e0009633. doi: 10.1371/journal.pntd.0009633 (PMC8336846; doi:10.1371/journal.pntd.0009633)
Supplement: Attachment — Submitted filename: Response to Reviewers.docx [file pntd.0009633.s002.docx]

Dear Editors，

Sincerest thanks for your response and reviewers’ comments on our manuscript entitled “ *Identification of potential biomarkers in dengue via integrated bioinformatic analysis*” (PNTD-D-20-02220R1). We have carefully studied the comments made by the reviewers and amended the manuscript. We would like to resubmit it for your consideration. And the revised manuscript has been submitted. Point by point responses to the reviewers’ comments are listed below this letter.

We hope that the revised version of the manuscript is now acceptable for publication in your journal.

I am looking forward to hearing from you soon.

With best wishes,

Yours sincerely,

Xu-Guang Guo.

We would like to express our sincere thanks to the reviewers for the constructive and positive comments.

**Replies to Reviewer 2:**

1. **Are the objectives of the study clearly articulated with a clear testable hypothesis stated?**

**-Yes. The objective of the study is to identify dengue infection biomarkers. In order to do so the authors analyze public data with a standard methodology.**

**Answer:** Thank you very much for your comments and recognition.

2. **Is the study design appropriate to address the stated objectives?**

**-Yes, it is. By analyzing available transcriptomes, filtering DEGs, proceeding with pathway enrichment, protein-protein interaction and then looking for miRNAs, the authors are able to find potential biomarkers for dengue infection.**

**Answer:** Thank you for reading and commenting carefully.

3. **Is the population clearly described and appropriate for the hypothesis being tested?**

**-Yes, the authors specify all of it in the manuscript.**

**Answer:** Thank you very much for your comments and recognition.

4. **Is the sample size sufficient to ensure adequate power to address the hypothesis being tested?**

**-Yes, there is enough data from these datasets to account for any statistical analysis.**

**Answer:** Thank you very much for your comments and recognition.

5. **Were correct statistical analysis used to support conclusions?**

**-Yes, the packages and tools the group used are standard and widely accepted in the bioinformatics' field.**

**Answer:** Thank you very much for your comments and recognition.

6. **Are there concerns about ethical or regulatory requirements being met?**

**-No, as they are using public data there is nothing to worry about.**

**Answer:** Thank you very much for your comments.

**Results**

7. **Does the analysis presented match the analysis plan?**

**-Yes, it does.**

**Answer:** Thank you very much for your comments and recognition.

8. **Are the results clearly and completely presented?**

**-Yes, they are.**

**Answer:** Thank you very much for your comments and recognition.

9. **Are the figures (Tables, Images) of sufficient quality for clarity?**

**-Yes, yes. The .tiff quality is excellent and the figures are disposed in an organized and clear way.**

**Answer:** Thank you very much for your comment and recognition.

**Conclusions**

10. **Are the conclusions supported by the data presented?**

**-Yes, they proposed to find biomarkers for dengue infection and so they did.**

**Answer:** Thank you for reading and commenting carefully.

11. **Are the limitations of analysis clearly described?**

**-Yes, the authors perfectly describe it in the manuscript. Even though there are no in vitro nor in vivo experiments, and this may limit their findings, this is discussed and accounted for. Nonetheless, these findings are a great contribution for future studies and may be helpful for researches in dengue therapy.**

**Answer:** First of all, thank you very much for your careful reading and comments. Indeed, without any financial support, it is difficult for us to carry out any in vivo or in vitro experiments to verify it. However, we also hope to make some contributions to the future research on the treatment of dengue fever.

12. **Do the authors discuss how these data can be helpful to advance our understanding of the topic under study?**

**-Yes, they state it clearly to the readers.**

**Answer:** Thank you very much for your comments and recognition.

13. **Is public health relevance addressed?**

**- Yes, identifying potential biomarkers for disease treatment (and in this case, infection control) are of extreme importance. Even more when talking about dengue, its relevance goes without saying given the number of infected people yearly.**

**Answer:** Thank you very much for your careful reading and comments.

**Summary and General Comments**

14. **Use this section to provide overall comments, discuss strengths/weaknesses of the study, novelty, significance, general execution and scholarship. You may also include additional comments for the author, including concerns about dual publication, research ethics, or publication ethics. If requesting major revision, please articulate the new experiments that are needed.**

**-The authors did a great work with the revision. This sort of bioinformatics analysis is of utmost importance for identifying therapeutic targets and controlling diseases. There is one point I would like to address, though. In your first submission we discussed about different infection's stages and how it may affect the patients' transcriptional profile. I don't know about limma, but when analyzing RNA-seq with DESeq2 there is a test called Likelihood Ratio Test (LRT) that may be used (instead of the default wald test) to identify genes whose expression change overtime. There may be a similar or corresponding test that can be used on limma and may prove to be useful for your future analysis.**

**Answer:** First of all, thank you very much for your comments and recognition of our efforts. As you mentioned, when analyzing RNA-seq with DESeq2 there is a test called Likelihood Ratio Test (LRT). However, we are not familiar with it, bu we searched the relevant study^1^ and learned that DESeq2 is a method for difference analysis of count data, which uses the shrinkage estimation of discrete and fold changes to improve the stability and interpretability of the estimation. It can be used for quantitative analysis of comparative RNA-seq data. Therefore, we all considered that the Likelihood Ratio Test provided by DESeq2 is of great significance for the differentially expressed analysis of RNA-seq data. In the future research, we will learn and understand this knowledge more deeply. As for the *limma*^2^, it is often used as a popular choice for differential expression analysis of microarray and high-throughput PCR data, as a particularly powerful tool for reading, standardizing and exploring such data. On the one hand, it fits a linear model for each row of data, and takes advantage of the flexibility of these models in various ways, such as dealing with complex experimental designs and testing very flexible hypotheses. On the other hand, it uses the high parallelism of genomic data to borrow the strength between gene models, allows different levels of variability between genes and samples, and makes the statistical conclusion more reliable when the number of samples is small. The data we included in our study is microarray data. Because it is measured as intensity, we need to carry out background correction and standardization before any statistical analysis. *Limma* includes a series of background correction and standardization programs for different types of microarrays. Noteworthy are the maximum likelihood implementation of the normal-exponential convolution model for background correction and the implementation of loess curves and normalization using quantitative weights. We added brief interpretation about *limma* package in the *Data processing* section of *Materials and Methods*.

1. *Love MI, Huber W, Anders S. Moderated estimation of fold change and dispersion for RNA-seq data with DESeq2. Genome Biol. 2014;15(12):550. doi:10.1186/s13059-014-0550-8*

2. *Ritchie ME, Phipson B, Wu D, et al. limma powers differential expression analyses for RNA-sequencing and microarray studies. Nucleic Acids Res. 2015;43(7):e47. doi:10.1093/nar/gkv007*

**Replies to Reviewer 3:**

**Methods**

**-Are the objectives of the study clearly articulated with a clear testable hypothesis stated?**

**-Is the study design appropriate to address the stated objectives?**

**-Is the population clearly described and appropriate for the hypothesis being tested?**

**-Is the sample size sufficient to ensure adequate power to address the hypothesis being tested?**

**-Were correct statistical analysis used to support conclusions?**

**-Are there concerns about ethical or regulatory requirements being met?**

1. **As per the authors reply over the reviewer comments, they are acceptable**

**Answer:** Thank you very much for your careful reading and comments.

**Results**

**-Does the analysis presented match the analysis plan?**

**-Are the results clearly and completely presented?**

**-Are the figures (Tables, Images) of sufficient quality for clarity?**

2. **Most the author reply is acceptable, except figure 1 explanation**

**The threshold was set as followed: P<0.05 and |log2FC|≥2. FC: fold change**

**Review comment by author: However, considering that it can ensure significant differences and screen out more differential genes for further analysis and comparability before datasets, this study took p < 0.05 and FC ≥ 1.5 when analyzing each dataset. Because when fold change took 2, the number of DEGs obtained from individual datasets are relatively small.**

**This both statements contradicts please clarify.**

**Answer:** For the explanation of Figure 1, we may have some problems in expression. We hope to further explain this. To select differentially expressed genes (DEGs) in microarray datasets with two biological conditions, we used fold change (FC), which was calculated as the ratio of the average values of control and test samples. Regarding the selection of fold change value, we have searched the research related to bioinformatics analysis, and found that different articles have different FC values. For example, the screening threshold of some studies^[1][2]^ are p-value < 0.05 and fold change ≥4 (log2FC≥2), some studies^[3][4]^ are p-value < 0.05 and fold change ≥ 2 (log2FC ≥ 1), some studies^[5][6]^ are p-value < 0.05 and fold change ≥ 1.5 (log2fc ≥ 0.58), and even some studies’ cut-off values^[7][8]^ are P < 0.05 and log2FC > 0.5. Then, in one study^[9]^, the researchers used different FC values as the screening threshold, and reported the DEGs, which showed that when FC value was > 1.5, more DEGs could be obtained than when FC value was > 2. Therefore, to sum up, the screening threshold of our study is selected as p-value < 0.05 and fold change ≥ 1.5 to obtain more DEGs for analysis.

[1]*Yang D, Li N, Ma A, et al. Identification of Potential Biomarkers of Polycystic Ovary Syndrome via Integrated Bioinformatics Analysis. Reprod Sci. 2021;28(5):1353-1361. doi:10.1007/s43032-020-00352-x*

[2]*Chen B, Ding P, Hua Z, Qin X, Li Z. Analysis and identification of novel biomarkers involved in neuroblastoma via integrated bioinformatics. Invest New Drugs. 2021;39(1):52-65. doi:10.1007/s10637-020-00980-9*

[3]*Chen Y, Liu D, Liu P, Chen Y, Yu H, Zhang Q. Identification of biomarkers of intrahepatic cholangiocarcinoma via integrated analysis of mRNA and miRNA microarray data. Mol Med Rep. 2017;15(3):1051-1056. doi:10.3892/mmr.2017.6123*

[4]*Liu J, Wu S, Xie X, Wang Z, Lei Q. Identification of potential crucial genes and key pathways in osteosarcoma. Hereditas. 2020;157(1):29. Published 2020 Jul 14. doi:10.1186/s41065-020-00142-0*

[5]*Yu S, Wu Y, Li C, et al. Comprehensive analysis of the SLC16A gene family in pancreatic cancer via integrated bioinformatics. Sci Rep. 2020;10(1):7315. Published 2020 Apr 30. doi:10.1038/s41598-020-64356-y*

[6]*Lu X, Ye K, Zou K, Chen J. Identification of copy number variation-driven genes for liver cancer via bioinformatics analysis. Oncol Rep. 2014;32(5):1845-1852. doi:10.3892/or.2014.3425*

[7]*Li Y, Tan W, Ye F, et al. Identification of microRNAs and genes as biomarkers of atrial fibrillation using a bioinformatics approach. J Int Med Res. 2019;47(8):3580-3589. doi:10.1177/0300060519852235*

[8]*Chen X, Wang J, Peng X, et al. Comprehensive analysis of biomarkers for prostate cancer based on weighted gene co-expression network analysis. Medicine (Baltimore). 2020;99(14):e19628. doi:10.1097/MD.0000000000019628*

[9]*Michel L, Reygagne P, Benech P, Jean-Louis F, Scalvino S, Ly Ka So S, et al. Study of gene expression alteration in male androgenetic alopecia: evidence of predominant molecular signalling pathways. The British journal of dermatology. 2017; 177: 1322-1336. https://doi.org/10.1111/bjd.15577*

**3. Figure 4 and 5 quality is poor.**

**Answer:** We are very sorry for the quality of Figure 4 and 5. And we have uploaded them again, hoping to meet your requirements.

**Conclusions**

**-Are the conclusions supported by the data presented?**

**-Are the limitations of analysis clearly described?**

**-Do the authors discuss how these data can be helpful to advance our understanding of the topic under study?**

**-Is public health relevance addressed?**

**4. The authors reply is acceptable.**

**Answer:** Thank you very much for your comments and recognition.

**Summary and General Comments**

**-Use this section to provide overall comments, discuss strengths/weaknesses of the study, novelty, significance, general execution and scholarship. You may also include additional comments for the author, including concerns about dual publication, research ethics, or publication ethics. If requesting major revision, please articulate the new experiments that are needed.**

**5. The authors reply is acceptable**

**Answer:** Thank you very much for your careful reading and recognition.

We tried our best to improve the manuscript and made some changes in the manuscript. We appreciate for Editor/Reviewers’ warm work earnestly, and hope that the correction will meet with approval. Once again, thank you very much for your comments and suggestions.
